# On the Anatomy of the Breast

**Published:** 1840-04-01

**Authors:** 


ON THE ANATOMY OF THE BREAST. By Sir *Astley Paston Cooper*, Bart. F.R.S. D.C.L. G.C.H. Serjeant-Surgeon to the Queen, Consulting Surgeon of Guy's Hospital, Member of the National Institute of France, &c. &c. Quarto, pp. 193. London, Longman and Co. 1840.

WE cannot avoid transcribing the Dedication of this splendid work. It places in so favourable a view the head and the heart of its author, that none who read it would wish it unread again.

" *To the Members of the Medical Profession.*

My Dear Brethren,—I dedicate this work to you for two reasons.

First: To express the delight I feel at observing your increased love for the Science of the Profession, and your earnest desire to found your practice on an intimate knowledge of Anatomy, Physiology, and Pathology.

Secondly: To thank you for your unwearied kindness and attention to myself during a period of fifty years.

Should I by this work add anything to your knowledge of the Anatomy of the Breast, I shall have received the utmost and only reward which I am anxious to obtain by its publication.

With every wish for your prosperity, I have the pleasure to remain,

Yours, most sincerely and gratefully,

ASTLEY PASTON COOPER."

*Conduit Street, January,* 1840.

Perhaps no man in our profession has ever occupied so enviable a position as Sir Astley Cooper does at present. By force of an industry which never tired, of talents well suited to the science which he chose, of physical accomplishments which could execute all that genius prompted, and were excellently qualified to win the admiration which a noble bearing extorts even from judgement and from age, Astley Cooper attained, and kept so long as himself could wish, *the first* place as an English Surgeon. Rich in wealth and in the world's regard, full of years and honour, the ambition of place and of gold contented, Sir Astley Cooper looked for something higher, more remote, more difficult to reach, and more lasting than money or popular esteem or any current distinction—he looked for the reputation of benefitting the profession and advancing the science that he loved. He retired from the active pursuit of practice, and commenced that series of investigations which have produced the splendid works on the Structure and Diseases of the Testis, the Thymus, and the Breast. These investigations have been conducted in the liberal manner which can alone, in these days, command success: time, and trouble, and pecuniary expenditure being held of no account, and lavished profusely on them.

To these works, composed in the evening of his days, Sir Astley Cooper may turn with an honest pride, and regard them as the sureties of posthumous fame. The desire of success may have prompted his earlier exertions, and the treatise on Fractures and Dislocations was the creature of experience that Fortune might have given. But that "age of ease" which is held up to our natural aspirations, is voluntarily broken in upon by Sir Astley Cooper, and turned to a period of activity and of toil as unremitting and productive, as if life were only just begun, and fortune and a Baronetcy in the perspective. Possessed of all that men most covet, of rank, property, reputation, a vigorous constitution, which unites the buoyant spirit of youth with the sober tranquillity of age, Sir Astley Cooper is an eminent object of attention and of imitation. Few can expect such fortune—all *may* emulate his zeal—and all *will* own his merits.

How true are the following remarks, how characteristic of the man, how simple an explanation of his success.

"I have restricted myself to describing from my own preparations only; and if every author in our profession would adopt this plan, and merely write on what he is capable of demonstrating, preserving, and exhibiting to others, the medical world would not be overwhelmed with those crude opinions, theories, and conjectures, which, according to the present system of quoting all that has been written, are sure to compose the greater part of the works that issue from the press. My rule has been to publish that only which I could show to those who were sceptical, and were yet desirous of arriving at the truth. The preparations delineated in my works on Hernia, on Dislocations and Fractures, on the Breast, on the Testis, and on the Thymus Gland, are all in good preservation, and can be immediately referred to, as they are either deposited at St. Thomas's or Guy's Hospital, or in my private collection. The parts represented in the present Description of the Structure of the Breast, are all in my own house.

Let all who labour in the Science of the Profession adopt this method, and we shall soon have numerous and useful specimens of normal and of morbid structures. Such preparations, collected both by private teachers, and by the medical persons attached to our different hospitals, will form a basis of valuable and undeniable publications for the present generation and for posterity." vii.

The following are Sir Astley's directions for preparing the human mamma for demonstration. First, it is necessary that the breast employed for the purpose should be that of a woman who has been for some time suckling: for the breasts of a woman who dies of puerperal fever, in the first few days or weeks from her confinement, are not in a fit state to be clearly developed, as the milk cells are not completely evolved, and the gland is so loaded with blood, that the ducts and glandules are obscured in it, not only at the time of injection and dissection, but when afterwards dried.

Secondly.—It is usual to inject the ducts with quicksilver; but this injection, although it answers well in displaying the milk cells, yet does not succeed in distinguishing the various lactiferous tubes of the different parts of the gland; and in the subsequent dissection, it is scarcely possible to avoid cutting the ducts, emptying the gland, and spoiling the preparation, however previously beautiful.

It is, therefore, better to inject the gland with size of different colours, or with coloured wax, by which at once each duct is distinctly shown, and evn the cells will be displayed.

The various ducts are so interwoven and intermixed with each other, that they can by these means only be distinguished, or their distribution be clearly demonstrated. Quicksilver gives a general idea of the structure, but coloured injections a clear, distinct, and very intelligible view of the whole; and the dissection may be readily conducted, without injury to the preparation.

Thirdly.—To ascertain the quantity of glandulous matter, at different periods of life, it is requisite that the breast be put for a short time in boiling water, when the skin and fat become detached, and the gland, like other albuminous compositions, is left extremely hardened, and perfectly insulated and separated from the surrounding parts. This process furnishes an opportunity of giving an estimate of the quantity of gland, at puberty, in the adult, and in old age; as will be seen in one of my plates. Dried, after being boiled, the gland may be preserved for many years.

Fourthly.—To unravel the milk ducts, and to demonstrate the fibrous tissue of the gland, it is to be macerated in warm water, and dissected from day to day; and its ducts and glandules will be separated and shown. A section of the breast should also be made, from the nipple to the pectoral muscle, and then macerated in warm water, and daily dissected, when the ducts, the secretory structure, and fibrous suspensory tissue, will be shown, between the gland and the skin in the interior of the organ, and in its passage to the aponeurosis of the pectoral muscle.

Fifthly.—To show the connexion of the breast with the fascia of the thorax, the axilla must be carefully dissected in the adult, and its fascia traced.

Sixthly.—The arteries, veins, and absorbents, must be minutely injected. These vessels are large at the period of lactation, but small before and after that process, and are then injected with difficulty.

Lastly.—To trace the nerve in the mammary gland, and in the nipple, the arteries must be previously injected with a fine injection, properly coloured, for if this be not done, it is impossible to distinguish their minute divisions from the finer branches of the arteries.

### *Form and Situation of the Breast.*

The breasts, or mammary glands, are composed of two sets of parts—the *external* and the *internal.* The external comprise the nipple, or mamilla, areola, tubercles, and some glands. The internal is the secretory organ, made up of an assemblage of small secreting bodies, or glandules, from which proceed the excretory vessels, or lactiferous tubes, to the nipple. The glandules and ducts are united to each other, by means of a *fibrous* and inelastic membrane, which penetrates the surface of the gland, and sends fibres into all its interstices, and by uniting its small constituent glandulous bodies, forms it into what is called a conglomerate gland.

A cellular membrane, both reticulated and adipose, also enters into the composition of the gland; and in this membrane not only is abundance of fat deposited, but also the arteries, veins, absorbents, and nerves hold their course, and are distributed to the substance of the gland, and to its appendage the nipple.

"The breasts are slung upon the chest, supported by the fibrous tissue, and they are projected at the nipple *forwards* and *outwards*. I have, in my work on the Testis, pointed out the errors of those who paint or chisel from imagination, and not from observation of nature, in placing those bodies of equal height, although the left is usually much lower than the other; and the same remark may apply to the breasts; modellers, sculptors, and painters sometimes represent the nipples as being pointed forwards, and place them as their imagination leads them to conceive them to be, and not as they really are. It is modern artists who fall into this error, for the ancients modelled from the living subject, and gave accurate representations of nature." 11.

This obliquity of the nipple is a beautiful provision of nature, as when the child rests upon her arm and lap in the most convenient position for sucking, for if the nipple and breast had projected directly forwards, the child must have been supported before her by the mother's hands in a most inconvenient and fatiguing position, instead of its reclining upon her side and arm. But it is wisely provided by nature, that when the child reposes upon its mother's arm, it has its mouth directly applied to the nipple, which is turned outwards to receive it; whilst the lower part of the breast forms a cushion upon which the cheek of the infant tranquilly reposes.

The following observation is important:—The margins of the breast do not form a regular disk, but the secreting structure often projects into the surrounding fibrous and adipose tissue, so as to produce radii from the nipple of very unequal lengths, and a circular sweep of the knife cuts off many of its projections, spoils the breast for dissection, and in surgical operations leaves much of the disease unremoved.

Sir Astley believes that the existence of two mammæ is a provision against disease or accident. He refers to the case of four mammæ related by Dr. Lee.

Frequent lactation, says Sir Astley, even in our own climate, leaves the breasts relaxed and pendulous, and alters both their form and their direction; it is, therefore, right that they should have a sling under them, a cushion, or stays to support them, to prevent their undergoing a change, which may by care, be, in a great degree, obviated. But it is the influence of warm climates which relaxes them most, and hence the women of the East and West Indies, who have had several children, have their breasts hanging to the upper part of the abdomen, suspended by a thin portion of skin from the part at which they originally grew. This relaxation allows them to suckle over the shoulder; the child being suspended from the back, elevates the breast to the clavicle, or if the breast be carried into the axilla, the child can suck under the arm, if the latter be raised. The Hottentot women make a shelf of their enormous nates for the child to rest on, while it sucks the breast slung, like a bag, over the shoulder.

It is of importance to be acquainted with the natural *feel* of the breast at different periods of life.

"The sensation imparted to the hand in feeling the breast, at different periods of life, very considerably varies. At the age of puberty, and for many years afterwards, the breast is dense, compact, smooth, and equal; but so soon as they become employed in lactation, they begin to separate into small bodies with indentations around them, and this arises from the stretch and relaxation of the uniting cellular and fibrous membrane. Even in single or childless women the breasts, towards the cessation of the sexual secretion, become often exceedingly lobulated. In age the lobulated feeling ceases from the absorption of the glandular structure. The return of the menstrual secretion also makes a great difference in the feel of the breasts, as they then become full, tense, and painful, and an ecchymosis sometimes appears. It is of importance to know these changes, as they lead to a clearer diagnosis in disease." 20.

*Structure and Constitueut Parts of the Breast.*

Sir Astley proceeds from without inwards.

*The Nipple or Mamilla.*—It is situated nearer the abdominal than the clavicular margin of the gland.

Its form is that of a cone, rather rounded at its extremity in the virgin, but it forms a flat surface in the lactating woman, the centre of which is cribriform, being perforated by the numerous terminations of the lactiferous tubes, which are placed in a cleft before lactation, but are spread out upon the surface pending that period.

In the child, continues Sir Astley, the nipple scarcely rises above the skin of the areola, but it usually grows to the age of puberty; in the adult female it is from half an inch to three quarters in length; in lactation it is often an inch. After fifty it remains elongated, but relaxed; in old age it is sometimes in a great degree absorbed, and when thus wasted, appears as a mere wart.

The nipple is nearly smooth until puberty. At fifteen years it has a cleft near its centre, in which are the orifices of the lactiferous tubes, and it forms an uneven hemispherical projection.

At sixteen years it is slightly wrinkled; at seventeen it has small papillæ upon its surface. From twenty to forty years the papillæ are large; from forty to fifty the nipple becomes wrinkled; from fifty to sixty the nipple is elongated, and in old age it usually has a warty appearance.

The colour of the nipple varies at different periods of life, and under different states of the uterus. In infancy it is of a pinkish-red; at puberty of a more florid red. In young women of a slightly brownish red, but in pregnancy it becomes of a very dark colour. In old age it becomes again more of the hue of the surrounding skin, although it sometimes remains very dark. The nipple is often defective, or buried in a cleft, and is sometimes entirely wanting; the first makes nursing difficult, and the second prevents it altogether.

The nipple is made up, *first*, of the common integuments—*secondly*, of the fascia covering and surrounding the lactiferous ducts—*thirdly*, of the milk tubes—*fourthly*, of the common organization of arteries, veins, absorbents and nerves—*fifthly*, of cellular tissue, in which those parts are found.

*The Cuticle* covers the nipple and projects between its folds and into its depressions. It sends processes into the lactiferous tubes, which processes may be drawn out after continued maceration. In women of light complexions, and more especially those with red hair, the cuticle is extremely thin, and is frequently subject to abrasion from the application of the child's lips in sucking, and the process of nursing is, from this cause, rendered exceedingly painful. Astringent applications, or even a shield, become necessary. Incrustations often form on the nipples of girls, covering their clefts and points, and requiring attention to prevent ulceration, which the unguentum hydrargyri nitratis, or unguentum zinci, are most fitted to oppose. In age an incrustation of a much firmer kind fills the cleft and covers the point of the mamilla.

*The Rete Mucosum* is not so abundant on the nipple as on the areola. It enters, with the cuticle, into the lactiferous tubes, and, in the larger mammalia, may be seen terminating, by a fringed edge, at a few lines from their extremities.

Some follicles exist in the nipple, and admit the cuticle and rete mucosum.

*The Cutis* of the nipple, in the state of lactation, is divided into two surfaces; the first forming the disk or circumference of the nipple, and the second its truncated apex.

The disk is composed of a great number of papillæ, which produce a vascular and sentient surface, and which form its erectile and highly sensitive tissue.

The direction of these papillæ is from the base towards the apex of the nipple, so that they are pushed back as the mamilla enters the mouth of the child, and thus greater excitement is produced. We need not dwell upon their form. They are very numerous and large, and highly vascular.

Erection of the nipple, says Sir Astley, has been supposed to arise from the passage of the blood into an elastic, cellular structure, like the corpora cavernosa penis, but there is no such formation in the nipple. It is a state arising simply from the determination of blood into the little bushes or assemblage of capillary arteries in the nipple and papillæ. The blood is propelled forwards to the papillæ by the action of the heart and arteries, so that by this vis a tergo, the capillary arteries become extremely distended, and erection is produced; it more slowly escapes through the little branches of communication with the veins, and which are more distant from and less under the influence of the vis a tergo from the heart, which is the principal source of the circulation; thus a congestion of arterial blood is produced in the capillary arteries. But when the excitement subsides, the blood is no longer directed with the same impetuosity upon the papillæ, and the veins will then remove the congestion in the extreme branches of the arteries, as the vis a tergo has in a considerable degree subsided.

In lactation, the orifices of the lactiferous tubes terminate in a kind of cribriform net-work, between the meshes of which the milk escapes. This net-work being very little elastic, yields but slightly to the pressure of the milk, so that the orifices of the ducts continue of very diminutive size, not only in woman, but in other animals: thus it is that the escape of the milk is prevented, excepting under excessive distention and in the process of suckling. The tubes, internally, are wrinkled longitudinally.

A fibrous tissue, or fascia, internal to the cutis, connects the nipple to the gland of the breast. The cellular tissue of the nipple is reticular, not adipose.

Of the *arteries, veins, absorbents,* and *nerves,* we need not speak, although they are described with admirable precision.

"In addition to the structures which I have described, there are, at the apex of the nipple, the numerous and minute orifices of the lactiferous tubes, which amount to more than twenty orifices when in great numbers, and from twelve to fifteen in others, but I cannot be sure that all the openings are lactiferous ducts, as some may be follicles only." 35.

*Of the Areola.*

The circle of skin which surrounds the base of the nipple, smooth until puberty, when it has little eminences and tubercles upon its surface.

The diameter of the areola in a child is about half an inch. At puberty, and in young women, it is an inch; during lactation, it is two inches or more; and although, in after age, its colour diminishes, its diameter remains almost the same, excepting in very old persons, in whom it disappears.

In the infant it is of a pinkish red; at puberty, of a darker red; in lactation, it becomes of a very dark colour, approaching that of the negro skin; in age it remains dark, but in old age it sometimes loses its colour, which becomes like that of the surrounding skin. This change of colour is an useful sign of pregnancy; but Sir Astley has known a diseased and excited state of the uterus after marriage, when that organ had become enlarged, but not impregnated, produce a swelling of the breasts, and a discoloration of the areola.

Sir Astley gives the following simple explanation of the darkening of the areola in pregnancy.

"The quantity of rete mucosum secreted must depend greatly upon the quantity of blood determined to the part. As soon as the influence of the uterus and ovaria is felt by the breasts, and they swell from more blood being determined to them, the rete mucosum is more largely secreted, and the colour of the areola and nipple becomes darker.

When the pregnant state of the uterus enlarges the breast, by increasing the flow of blood to it, the rete mucosum increases in quantity; but still more in lactation, when the nipple and areola are greatly excited, the depth of colour is the greatest, and the best opportunity is afforded of observing the colouring matter.

As the circulation declines in age, the rete mucosum diminishes in the areola.

The menstrual secretion has, from the change thus produced upon the breast, some influence upon the colour of the nipple and areola."

The *Cutis* of the Areola presents papillæ, like those of the nipple, but smaller. Their apices are directed towards the nipple, so that they are opposed to the papillæ of the lips of the child. Their use is three-fold. First, they give a greater adhesion to the infant's lips in sucking; and secondly, they add to the sensibility and sympathies of the areola with the mammary gland; thirdly, they form a surface which is embraced by the child, and received into its mouth, so that the large lactiferous tubes behind the areola are emptied by the pressure of the lips of the infant.

The areola is, therefore, to be considered as an extension of the nipple, the base of which latter is lost in the former.

*The Tubercles, or Mucous Glands of the Areola,* are very perceptible at the base of the nipple, and upon the surface of the areola. Their orifices, in number from one to five, are visible to the naked eye. Sir Astley proves them satisfactorily to be mucous glands, which perform these offices:—first, they discharge from their little springs a lubricating secretion; secondly, they add to the firmness of adhesion of the child's lips; and thirdly, they give greater sensibility to the areola, and sympathetically excite a larger secretion from the mammary gland.

They are much enlarged in lactation, and pour out a fluid, which is coagulated by alcohol, and its appearance is like that of white of egg. The fluid they secrete has a tendency to lessen that excoriation which, when it does occur, renders suckling almost an agony. They are extremely vascular, lobulated, and cellular. Each orifice opens into an arborescent vessel, or vessels.

On the general surface of the *Skin of the Breast,* are a number of *Sudatory Glands*—from which much perspirable, and mucous matter can be squeezed; and from, or near them, small hairs proceed.

### *On the Internal Parts of the Breast or Mammary Gland.*

The parts which enter into its composition are:—*First,* the fascia mammæ. *Secondly,* the lactiferous tubes, or milk ducts. *Thirdly,* the glandules in which the milk is secreted. *Fourthly,* the milk cells. *Fifthly,* the common organization of arteries, veins, absorbents, and nerves. *Sixthly,* the fat and cellular tissue.

*The Fascia Mammæ.*—It forms a superficial and a deeper layer, including the gland of the breast between them, and starting outwards from the ligamentous substance covering the sternum.

The anterior or superficial layer passes upon the anterior or cutaneous surface of the breast: here it forms a fibrous covering, but not a true capsule, spread upon the surface of the gland, and passing between the gland and the skin; but it also enters the interior of the secretory structure.

Here it sends out two sets of processes of a fibrous nature from its two surfaces.

Anteriorly, large, strong, and numerous fibrous or fascial processes, to the posterior surface of the skin which covers the breast, into the substance of which it is received, and with which it is incorporated.

It is by these processes that the breast is suspended in its situation. Sir Astley, therefore, calls them the *ligamenta suspensoria.*

When the breast is placed in its natural position, the posterior extremities of the ligamenta suspensoria are spread over the fore-part of the gland, support numerous folds of the glandular structure, penetrate the substance of the organ, and everywhere connect the portions of glands to each other. A process proceeds to the nipple, and, as has been stated, connects it with the gland. Between the ligamenta supensoria, the lobes of fat are placed, which serve further to defend this organ from injury.

The posterior layer of the fascia, when it has reached the margin of the gland, passes behind it, and sends forth two layers of fibres. The anterior of these fibres pass on the back of the gland, sending processes of fascia into the organ to unite its parts, and other fibres which pass from one ridge of the gland to the other posteriorly, giving it a smoother surface than that of the anterior part of the breast, as it is not folded in the same manner.

The other fibres of this deeper seated fascia pass backwards, and are united to the aponeurosis of the pectoralis major.

Thus then, sums up Sir Astley, the breast is supported by the two portions of fascia; the superficial layer connecting it to the skin anteriorly, and forming the ligamenta suspensoria, and the posterior layer of fascia joining it to the pectoral muscle, by its aponeurosis; and between these two processes it swings, and yields to pressure and to violence. Whilst the fascia thus affords support, it also firmly unites the different portions of the gland to each other, throughout the whole of the substance of the organ, by entering into its interior composition.

*The Glandular Structure* of the mamma, traced from the nipple back into the gland, in a contrary course to that of the milk, is made up:—*first*, of the straight lactiferous tubes in the nipple, or the *Mammillary Tubes*. *Secondly*, of these tubes suddenly enlarged at the base of the nipple, and under the areola, and which contain a large quantity of milk; these are the *reservoirs*, or areolar tubes. *Thirdly*, of these tubes becoming arborescent in each part of the gland, and forming the mammary ducts. *Fourthly*, of *glandules*, disposed in lobuli, which constitute the principal part of the mammary gland, and from which the milk tubes originate. *Fifthly*, of the milk cells, into which the milk is first secreted by the mammary arteries.

*Of the Straight, or Mamillary Tubes.*—"The greatest number of lactiferous tubes I have been able to inject, has been twelve, and more frequently from seven to ten. But the greatest number of orifices I have been able to reckon has been twenty-two; however, some of these might have been follicles only, and not open ducts. I have had delineated two preparations of straight tubes, in one of which I found thirteen, and in the other twenty-two." 53.

Their size varies, some of the orifices only admitting a bristle, while others are as large as a common pin.

"They commence in a cribriform surface formed by the skin, with some mixture of fibrous tissue; so that these orifices do not increase much, or yield to the pressure of the milk. A probe of large size will pass to their orifices, if introduced from the gland, but it cannot be made to escape through the orifice of the duct, without employing great force to overcome the resistance, and even to lacerate the orifice; in that respect resembling the urethra in the female, which will admit the little finger from the bladder, but only a probe at its orifice." 53.

When the mamillary or straight tubes have passed these orifices, they begin to dilate, and to assume a conical form, gradually increasing in diameter to the basis of the nipple, and are therefore much larger than at the apex of the mamilla.

They are surrounded and enveloped by the fibrous tissue which lines the nipple, and which sends fibres between the tubes to keep them in their situation, and to strengthen them, and prevent their laceration.

The branches of arteries, veins, nerves, and absorbents pass, in cellular tissue, between the ducts. The mamillary tubes, when cut open, are found lined with mucous membrane, wrinkled longitudinally, and highly vascular.

*The Areolar Portions of the Tubes, or Reservoirs* begin at the basis of the nipple, extend under the areola, and to some distance into the gland, when the breast is in a state of lactation.

Their greater size than that of the mamillary tubes is in part owing to the loss of the pressure of the nipple, but principally to the number of branches of milk tubes which enter them from the breast; five or six large branches are combined in a reservoir.

These receptacles are of a conical form, like the mamillary tubes; and they begin from the extremities of the larger branches of the milk tubes, and terminate in the straight ducts of the nipple.

The appellation of reservoirs is less applicable in the human female than in other animals, for example, the cow; in her, they are capable of retaining a quart of milk, or more.

In the human subject they generally radiate from the nipple, although some of them pass directly backwards to the posterior or pectoral surface of the gland.

Their calibre is out of all proportion larger than that of the straight or mamillary tubes, and much larger than that of the milk tubes, which form their continuations.

When cut open, the reservoirs are found to be lined with a very vascular mucous membrane, like the mamillary or straight ducts, but they have a fibrous coat upon the outer side of this, which preserves their form, and which gives them their power of resistance to the great dilatation which the milk would otherwise produce. Their use is to supply the immediate wants of the child when it is first applied to the breasts, so that it shall not be disappointed.

*The Mammary, Lactiferous, or Milk Tubes* begin from the glandules, or secretory structure, in small and numerous branches, and increasing in size, terminate in forming the reservoirs.

They divide into branches, which increase in number as they proceed from the centre to the circumference; and their general appearance when injected, resembles that of the root of a tree.

The radiations of one of the mammary tubes sometimes occupies one-sixth to one-fifth of the circumference of the breast. On the sternal and clavicular aspect of the breast, a single duct radiates to the margin; but upon the axillary and abdominal aspects, two or three ducts ramify to the circumference of the gland, so that two or three ducts are placed upon each other. Hence the greater thickness of the lower and outer part of the breast, which enables it to form a cushion, on which the cheek of the child reposes.

The branches of the ducts do not radiate equally to the circumference, for some are much longer than others, and are lost on the fascia which encircles the breast, rendering its margins unequal.

In other parts the ducts at the margin of the gland are turned upon the gland, so as to form a kind of hem at its circumference, and to produce also a thickening of the substance of the breast from this cause.

Many of the mammary tubes upon the anterior surface of the breast are turned forwards to the skin, and connected to it by the ligamenta suspensoria; so that in removing the skin from the fore part of the breast, many of them are necessarily divided.

The breast is not formed into regular lobes by the ramifications of the ducts, because they ramify between, and intermix with each other.

"The most simple idea," ingeniously continues Sir A. "which can be formed of the mammary ducts, especially at the lower and outer part of the breast, is, that supposing them to resemble the roots of trees, as they do, that one root is growing between others, destroying regularity, and distinctness of their growth. Or suppose one hand applied upon the back of another, and the fingers introduced between each other, and then the fingers of one hand inclined to the right, and those of the other to the left, it conveys the idea of the above-mentioned intermixture." 59.

On the posterior surface of the gland, which is more smooth than the anterior, the ducts ramify more equably.

The mammary ducts do not communicate with each other, as is easily shown by throwing injections of different colours into the ducts, or by injecting one duct only.

"I have only seen one instance to the contrary of this position, in injecting a milk tube from the interior of the gland towards the nipple, two large branches of ducts crossing each other, where they laid in contact, the injection found its way by rupture, or by a deviation from the natural structure, from the one into the other duct, of which I have given a figure; and as this has only occurred once in more than two hundred times, it shows that it is not the result of a common structure. In the cow, the goat, and the ewe, in which there are different glands terminating at each teat, in a single duct, when the injection is thrown into one teat, it does not escape into any other gland." 60.

After lactation, when the mammary gland is injected, the lactiferous or mammary tubes appear cellular, for wherever two or three large branches enter, a sudden increase of size is produced, so as to form a little pouch, open at each end. These dilatations are also seen during lactation, when two or three branches are received at any part of the ducts.

The mammary ducts are formed by a fibrous coat upon the outer side, and within, by a mucous membrane. The latter is highly vascular.

*Of the Gland.*—The mammary ducts begin directly from the glandular structure, in very fine and minutely divided radiated branches, and after becoming larger and larger as they approach the areola, they terminate in the reservoirs.

The gland is constituted by the union of a number of glandules, which are connected by means of the fibrous or fascial tissue of the gland.

When injected and unravelled, they appear of considerable size; but when further examined, these larger bodies are divided into small glandules.

Between these glandules, the mammary tubes may be observed to ramify, and from these bodies their branches directly spring.

When these glandules are filled with injection, and for a long time macerated in water, and unravelled, they are found to be disposed in lobuli; and when a branch of a mammary tube is separated, with the glandules attached, the part appears like a bunch of fruit hanging by its stalk.

The body of the gland is formed by the union of these little glands, everywhere interspersed through it, and united by fibrous tissue.

Their size depends upon the state of the breast; after puberty they exist, but are not easily separated or unravelled.

In lactation they are large, may be minutely injected, and distinctly developed. In age they diminish gradually, and after a time disappear, leaving the ducts still distinctly ramifying, but without the true glandular structure.

On the anterior surface of the breast, the glandules are drawn towards the skin by means of the ligamenta suspensoria, and form folds or loops, like the petals of flowers; a disposition which, without increase of bulk increases the secreting surface.

The margin of the gland is extremely irregular; for it forms numerous processes, which proceed into the surronnding fibrous and cellular tissue. The lower and outer aspects of the gland are so folded as to give additional thickness there, while the greater number of glandules there contributes to the same effect. But the posterior surface of the breast is not folded and looped up like the anterior; but the ducts and glandules are, in the larger part of this surface, disposed in ridges connected by a fibrous membrane, which mats them together, and enters between the ridges into the interior of the gland.

The breast then is made up of an assemblage of glandules, united by a fibrous tissue.

From the nipple the gland begins to form little petals like those of a blooming rose, and they are turned forwards to the skin, to which they are connected by the ligamenta suspensoria; and in the depressions between them, the fat is lodged.

The glandules vary in their size, from that of the head of a pin to the bulk of a small tare, when the breast is in a state of lactation.

Their figure is oval when they are uninjected, and they are more pointed at the extremity farthest from the nipple, than at the place at which the mammary duct enters them.

*Of the Milk Cells.*—When the lactiferous tubes are minutely injected, they are found to proceed from each glandule, and when an injection is made of the glandules with quicksilver, size, or wax, they will be seen to be composed, in their interior, of numerous cellules, which are the milk cells.

Their number is very great, and too variable to admit of counting.

Their size in full lactation is that of a hole pricked in paper by the point of a very fine pin; so that the cellules are, when distended with quicksilver or milk, just visible to the naked eye.

They are rather oval than round, being slightly elongated where the branch of the lactiferous tube springs from them; but they appear more rounded to quicksilver, and when distended with milk, than when filled with wax.

When well injected and dried, the glandules form a kind of foliage in the breast, and each leaf is filled with these cellules. In the fulness of lactation, these leaves are full of cells, which can be readily injected and demonstrated; but at other periods they do not admit of being filled, and a most minute injection may then be made of the lactiferous tubes, yet no cells appear. Sir Astley has seen the lactiferous tubes become cellular, as they spring from the milk cells, but only just at their commencement, and under very minute injections.

He thinks that the cells are lined with mucous membrane, partly from their vascularity, partly from analogy; for, in the larger animals, as in the cow and the rhinoceros, the mucous membrane lining the ducts has no break in it, but may be seen to be continued so far as the parts can be traced by the eye, and by magnifying powers.*

The milk cells possess a considerable degree of elasticity, but in the human subject less than in other animals.

The arteries become extremely minute on the glandules, and around the cells. There are veins, of course; and absorbents arise from the cells in great numbers. The smallest branches of the nerves accompany those of the arteries. The course of the milk may now be understood.

"It is secreted by the arteries into the *milk cells*, from which it is forced forwards by two causes; first, by the elasticity of the cells, which is proved to exist in many animals by injecting the cells minutely with quicksilver, and then if one of the ducts be pricked with a needle, all the lactiferous tubes become instantly emptied: but in woman this occurs less than other animals.

Secondly, by the vis a tergo of the continued secretion, one portion of milk forcing forward the other, in a minor degree when the child is not applied, but when the draught occurs, a sudden rush of blood increases the secretion, and rapidly hurries the milk forwards to the nipple, to supply the wants of the infant.

The milk is conveyed from the cells which are found in every point of the gland into the *mammary ducts*, which form radii, converging all of them towards the areola; and as these vessels are increasing in their diameters, little opposition is made to the progress of the milk, as it courses from the smaller to the larger tubes.

When the milk is thus brought by the mammary tubes to the areola, it is received into the *reservoirs*, and in these, and in the mammary ducts, it is retained until the infant begins to suck; and here it will be seen that the form of the tube is reversed, for the mammary tubes are constantly increasing towards the nipple; but the reservoirs are large towards the gland, and become smaller towards the nipple, which gives them a power of retention until the discharge of the milk is required.

The milk next passes into the mamillary ducts, or straight tubes of the nipple.

These, like the reservoirs, are conical, with the apex of the cone turned to the point of the nipple, and as their orifices at the nipple are very small and unyielding, the milk is also again retained until the act of sucking removes it; and when the draught occurs, abundance of milk is hurried forwards to the reservoirs and mamillary tubes.

The infant's lips and gums, and the suction produced by the exhaustion of the air in the mouth, not only mechanically empty the mamillary tubes, and overcome the resistance of their orifices, but also, by rendering them finer capillary tubes, assist, upon hydraulic principles, in giving rapidity to the passage of the milk." 70.

### *Of the Fat of the Breast.*

The adipose matter is abundant. It is intended, says Sir Astley, first, to preserve the contour of the organ, by filling up all the depressions between the glandules.

Secondly, to regulate the temperature of the gland under exposure, whether from the poverty which preludes the possession of proper covering, or the caprices of fashion, which forbid its being worn.

---

* "Also after the secretion of milk has ceased, the secretory structure is often loaded with mucus."

Thirdly, for the purpose of allowing the breast to float in an oily fluid, for the adeps is fluid in the heat of the living body; and the gland thus eludes the injuries to which it might otherwise be liable.

Fourthly, to defend it from, and to lessen the effects of violence upon the part, which heavy blows or falls might occasion.

In a large and fat person, the breast is far removed from the skin, and from the pectoralis major muscle, by the immense quantity of adipose matter placed before and behind the gland, and in the intervening structures.

On the anterior surface between the gland and the skin, we find the fat deposited in very large lobes between the ligamenta suspensoria and anterior folds of the glandular substance on every part of the breast, and it also exists between the layers of fascia beyond it.

It is formed by a vascular membrane of the cellular adipose kind. The thick cushion of fat placed under the skin enables women of the lower class to bear the severe blows which they very often receive. Sir Astley has seldom known them to suffer immediately any serious consequences. Thin women, however, will experience severe bruises, which, in a short time, disappear. Tumors in the breast are often imputed to blows received long before.

The fat is also deposited behind the breast, as before it.

When the period of lactation is passed, and the breast begins to be absorbed, fat is abundantly deposited, to fill up the deficiency of glandular matter, and to preserve the natural form of the part. But in very old age, both the gland and the fat become absorbed, and the chest is then flattened like that of the male.

Of the *Arteries* and *Veins* of the breast we need say no more than is contained in the following quotation:—

"As the vessels are extraordinarily increased in some malignant diseases, and excessively distended with blood, so as to produce much pain to the patient, from this accumulation and distension, in addition to other causes, I have for more than twenty years been in the habit of bleeding in these complaints, by opening the veins of the part. When the pain is severe, and the functions of the chest are embarrassed, it affords instantaneous and great relief.

For this purpose, I have always in my case, a needle which cuts upon each side, its edges being lancet-shaped, and after placing my finger on a large vein, formed by the junctions of several veins, and between the breast and the clavicle, I prick the vein with this instrument. A lancet would answer equally well; but it excites more apprehension on the part of the patient, and makes a larger wound.

The opening is much smaller than that produced in bleeding in the arm. The quantity of blood considerably exceeds that drawn by leeches; and it may be extracted in three or four minutes to the amount of from four to six ounces, and the surgeon avoids the long-continued exposure which the application of leeches requires, and the trouble and inconvenience of the continued fomentation afterwards." 82.

A piece of lint and adhesive plaister should be applied over the puncture.

The *Absorbents of the Breast* possess a high interest for the surgeon, being, as they are, the carriers of malignant depositions into the system.

Without entering into the minute anatomical details, for which, as for much else, we must refer to the splendid work itself—we must content ourselves with extracting the summary of the route which such malignant matters take.

When the axillary, or clavicular side of the breast is affected, the absorbent glands in the axilla which are immediately connected with the mammæ, are diseased; and next the absorbents from the arm, and their glands; and then the arm becomes greatly enlarged. The cervical and subclavian glands are involved in the disease, and the absorbent vessels behind the scapula are affected.

When the sternal side of the breast is diseased, two lines *may be traced of absorbent enlargement;* first, from the breast, sometimes to the first and second, at others to the second and third intercostal spaces; and secondly, to the fourth and fifth intercostal spaces between the cartilages of those ribs, and then the disease proceeds concealed behind the sternum, within the anterior mediastinum.

When the disease is seated in the posterior or costal surface of the breast, or when the axillary glands are much affected, the disease enters the chest through the intercostal muscles, and passes between the pleura and the ribs, often in its course affecting the pleura, and producing tubercles in it, and it excites inflammation of this membrane, so as to cause adhesion between the costal and pulmonary pleura, and these adhesions become also malignant.

Sir Astley has seen the pleura to great extent thus diseased, towards both of the mediastina, with some adhesion of the lungs, and where they did not adhere, accumulations of water had taken place in the cavity of the chest. And the absorbent vessels themselves will become morbidly changed and obstructed, the tubercles are enlarged just under, and sometimes in the skin, and they form hard and knotted swellings in the circumference of the nipple.

The *Nerves of the Breast* are derived from the second, third, fourth, fifth, and sixth dorsal nerves, but mainly from the fourth and fifth.

On the *Evolution of the Breast* we cannot touch, but must direct our readers to Sir Astley.

*Of the Effects of Gestation and Lactation on the Breast.*

The breasts at this time receive much larger quantities of blood, and they generally swell and become painful, feeling heavy.

The nipple grows, and its papillæ become foliated and protuberant.

The areola becomes darker in its colour, thicker in its substance, and its diameter increases from one to two inches.

The tubercles and glands of the areola and those of the surrounding skin of the breast are rendered much more distinct and prominent than before.

The gland is loaded with blood. The ducts are enlarged, and injectible, but not so the cellules. After a few weeks' suckling, the nipple becomes very large and truncated at its apex, so as to form a broad flat surface, upon which the orifices of the lactiferous tubes are evolved; and the areola, as well as the nipple, can be in a great degree drawn between the lips of the infant when it is sucking. Milk cells can be injected in all the glandules.

*Of the Milk.*—Passing over a capital account of cow's milk, we stop for a few minutes at a comparison of *human milk* with it.

When first drawn, it appears more blue than that of the cow. It has a sweetish, but also a saltish taste. Soon after it is drawn, like cow's milk, it changes, if it be at rest, by a mechanical separation, from the less specific gravity of cream; the cream separating upon its surface so that it divides itself into cream and milk, but with this striking difference, that the milk in the human subject appears semi-translucent like whey, instead of being white and opaque as in the cow, so that it may be almost said to divide into cream and whey.

*Of the Cream.*—Its specific gravity is 1·021. The quantity of cream is abundant, if the woman be healthy; but it varies according to the age of the child, the habits of life, the food, the health and tranquillity of mind of the mother. In these respects women widely differ from other animals. From experiments it would appear that the cream, in comparison with the milk, is from one-fifth to one-third, varying with the health, the food, the habits, and state of mind of the mother.

The quantity of cream also varies as the time elapses from the birth of the child. A woman who was very poor, and had an exfoliation of the os frontis—7 measures of milk gave only 1 of cream. A woman highly fed—9 measures of milk had $3\frac{1}{2}$ of cream.

The cream of human milk, agitated for a length of time, did not produce butter; but milk and cream mixed together produced, by long agitation, a white and soft solid, in small bodies, which became an aggregated white butter; but with difficulty, and after a length of time.

*Of the Curd.*—This appears less early than in cow's milk, but separates gradually after ten days or a fortnight, and continues to do so for a length of time.

*Sugar of Milk.* When the cream and curd are separated, the whey is found to contain abundance of saccharine matter, which is the sugar of milk.

The colostrum, or milk which is at first produced after parturition, appeared at first of a yellow colour, and thick consistence; but when it had stood twenty-four hours, it separated abundance of imperfectly-formed cream upon its surface. Nine measures gave 6 of cream and 3 of milk. The milk had a slight tinge of red, the cream a somewhat deeper tint.

The colostrum of the cow contains a great number of particles of various sizes, apparently made up of numerous cohering globules, so as to present an extremely granular appearance; these granular bodies, which are absent in ordinary milk, are completely soluble in ether, and consequently are composed almost exclusively of fatty or oily matter.

From an interesting chemical account of milk, furnished to Sir Astley Cooper by that talented young chemist, Dr. Golding Bird, we can only abstract the following concluding observations.

"Milk may be physiologically regarded as made up of three classes of ingredients; the first containing those which resemble vegetable secretions in the absence of nitrogen; the second including those which contain abundance of nitrogen, and consequently afford a proper pabulum for the growth of the young animal; the third class containing those ingredients which, in the present state of chemical physiology, we have no safe grounds for supposing are *digested*, or their elements re-arranged by vital chemistry, and hence differ from the first two classes in being rather *appropriated* by the vital influence of the infant animal, than assimilated to form such combinations.

A. *Ingredients of milk in which nitrogen is absent.* Sugar of milk, fatty matters.

B. *Ingredients of milk in which nitrogen is present.* Caseous matter.

C. *Inorganic or saline ingredients.* Salts of potass, soda, lime, and iron.

The latter class contains those earthy salts which constitute the chief ingredients in osseous structures; and all being dissolved in, or diffued through, abundance of water, become fitted to pass or drain through the minutest vascular tissues." 126.

*On Lactation.*

The secretion of milk commences on the third or fourth day after the birth of the child, but there is a fluid produced during the latter part of gestation, which is not true milk. The milk will continue to be secreted for many years.

The secretion may be *constant*, the production of the fluid being slow and continued; or *occasional*, when there is what is called by mothers and nurses, the *draught* of the breast, by which is meant a sudden rush of blood to the gland, during which the milk is so abundantly secreted, that if the nipple be not immediately caught by the child, the milk escapes from it, and the child when it receives the nipple is almost choked by the rapid and abundant flow of the fluid; if it lets go its hold, the milk spirts into the infant's eyes.

Even the sight of the child will produce this draught, or sudden rush of blood and copious supply of milk, as the thought or sight of food occasions an abundant secretion of the saliva.

The draught is also greatly increased by the child pressing the breast with its hands, by its drawing out the nipple by its tongue, lips, and gums, and by the pressure of its head against the breast.

In other mammalia, so far as we can judge, a similar process occurs, and the same effect is produced by the animal striking the udder with its head, and forcibly drawing out the teat.

The quantity of milk which can be usually squeezed from the mother is about two ounces from one breast, but necessarily varies with the state of the health and mode of nutrition; as to the quantity produced by the draught, there are no means of accurately ascertaining it.

A woman who milked her right breast for Sir A.'s information, and whose child was four months and a fortnight old, produced :—

| | | |
|---|---|---|
| On the | Saturday Morning . . . | 2 oz. |
| ,, | Sunday Morning . . . | 2 ,, 2 dr. |
| ,, | Monday Morning . . . | 2 ,, |
| ,, | Tuesday Morning . . . | 2 ,, 6 ,, |
| ,, | Tuesday Evening . . . | 1 ,, 3 ,, |

At seventeen months after delivery, a woman milked out 2 oz. when the child had been seven hours absent from the breast.

Sir A. has often had this experiment made, and has almost constantly found that the morning's milk is greater in quantity than that of the evening, and the same observation generally applies to the cow.

The secretion of milk will continue for many years in an healthy mother, if it be encouraged by the application of the child to the breast.

"A woman had abundance of milk at eighteen months after delivery; another suckled her child for twenty-one months, and the child had no other food. Mr. Wakefield, of Battle Bridge, Pentonville, told me that he knew a woman who had suckled her two successive children, at the same time, and I have heard of an instance in which a wet nurse suckled two consecutive children."

"Mr. King informed me that when travelling in the Arctic circle (?) he had seen an Esquimaux boy play out of doors with his bow and arrow, and come into a hut, to receive the milk of his mother's breast; and many children in our own country play about a room, and then run to their mother's breast, and sometimes fetch a stool to stand upon, whilst they pursue the process of sucking." 132.

If the mother wishes to wean her child, and she still secretes abundance of milk, the best mode of removing it is by giving an active purgative in the morning, and applying evaporating lotions.

"Some women are prevented from suckling by want of milk; some by want of strength; some from a deficiency of the nipple; but too frequently it is the result of caprice, the fear of trouble, the dread of spoiling the figure, and from anxiety to avoid the confinement which it enforces; and in some from the contrary desire of having many children.

However, it is quite true that there are women who are too feeble to continue to be nurses, after giving suckling a fair trial. It injures their digestive powers; they feel a sinking sensation in the stomach; loss of appetite; pain in the chest, back, and head; violent spasms from its influence upon the nervous system, and becoming emaciated, they are compelled to give up a duty which they have been most anxious to fulfil." 134.

In general, the milk of a woman who becomes pregnant disagrees with the child, or is actually refused by it.

"The quantity of milk which a woman is capable of secreting, cannot be estimated by the size of her breast, as it often is large and hard rather than secretory, or it is loaded with adeps, and produces but little milk. The same remark applies to quadrupeds, as the cow with the largest udder does not always give the most milk. I know a lady, who, before pregnancy, has scarcely any breast; but it evolves largely in lactation, disappears in a great degree when the child is weaned, and again evolves with the next child. Now that she has ceased to have children, her breast is as small as that of a man, so that the chest in that respect resembles that of the male."

"After the first few weeks of lactation, there is little difference in the milk of three, six, or nine months, as is proved by the children of wet nurses being older or younger than those they suckle, yet still the children they nurse are well nourished. The same thing is proved by nurses suckling consecutive children. Dr. Walshman and Dr. Key, two of the most experienced accoucheurs I have known, informed me that they did not believe that the age of the milk made any essential difference. However, there is a feeling upon this subject on the part of the mother, which may be indulged, as she and the nurse are better satisfied, if the children be nearly of the same age; and Dr. Merriman thinks that the child of the wet nurse should be about two months older than the new-born child." 135.

A child may be deprived of its mother's milk, and pine for her breast, and if returned to it after several weeks, the secretion of the gland will return, and the child be supported by it.

Sir Astley is a decided advocate for a woman's suckling, for, independently of the moral considerations which should weigh with all properly constituted minds, and of some medical arguments which cannot but be familiar to us, he urges the following strong one:—Suckling also diminishes the disposition to malignant diseases of the breast, for although women who have had children are still liable to cancerous and fungoid diseases, yet it is undoubtedly true that breasts which have been unemployed in suckling, in women who have been married, but are childless, and in those who have remained single, are more prone to malignant diseases that those of women who have nursed large families; and if it were only to lessen the probability of the occurrence of such horrible complaints and causes of dissolution, women ought not to refuse to suckle their offspring.

A woman who has children and suckles them, is undoubtedly a better insurable life than a married woman who has no children, or one who has remained single.

Sir Astley, also, dwells on the advantages to the child of sucking the maternal breast. Dr. Merriman informed him that he tried to ascertain the average mortality of children brought up by hand, but found it difficult for want of accurate data. The result was a conviction, taking the whole population of rich, the poor, and the middle classes, that not more than two in ten children so nourished, survived eighteen or twenty months, and the mortality of the children of those who go out as wet nurses is frightful.

*Food of the Mother or Nurse.*—This is an important point, and we quote freely from Sir Astley, in reference to it.

"It appears," he says, "that the quantity and quality of the food taken by mothers and nurses, is often greater than is absolutely necessary; indeed, absurdly and unnecessarily abundant. A mother who reared ten very healthy children and never failed in her milk, adopted the following plan of diet:—

Her breakfast was café au lait with bread and butter. At one o'clock P.M., she took hot meat, and drank half a pint of porter. At six o'clock, she dined plainly upon meat, but drank half a pint of porter and two glasses of port wine. At ten o'clock P.M., she took a slight supper of meat and drank half a pint of porter. She suckled early in the morning, frequently in the day, and the last thing at night. During the night, the child was fed upon barley-jelly, gruel, flour and milk, milk and arrow-root if the bowels were relaxed. Her general food for the child was flour tied in a cloth boiled in water, dried and grated into milk with sugar.

It appears, however, that this diet for the mother is unnecessarily abundant and stimulating.

The Welsh women live, whilst they suckle their children, upon barley-bread, oat-cake, cheese, and oatmeal, and bacon, with leeks, and other vegetables boiled together, into what they call cowl. No beer nor wine, but milk and water, or butter-milk, are their drinks. The woman is often moving about her house in the fourth or fifth day after parturition. They are affectionate mothers, and their infants are generally very healthy.

In Ireland, Dr. Woodroffe of Cork informed me, in reply to some questions I put to him:—

| QUESTIONS. | ANSWERS. |
|---|---|
| What is the diet of the *poor* women in Ireland, whilst they are *suckling?* | Potatoes, milk, stirabout, and occasionally a little fish. |
| What work are they called upon to do whilst they are nurses? | They work in their fields and gardens, and are engaged in their domestic concerns. |
| How long do they generally *suckle*, and do you know of any individual cases of the child continuing to be suckled for a long period? | Never less than *twelve*, but more generally for *sixteen* or *eighteen* months. I have known many instances of children being suckled for *two* years, much to the detriment both of mother and child. Amongst the lower classes, there is a strong prejudice in favour of weaning the child on particular days; and to accomplish this object, they often continue to nurse their child *five or six months* longer than they otherwise would. |
| Do they carry the child with them whilst at work, or do they go home to suckle? | The child is left at home; women do not go far from their own dwellings, but work in the adjoining fields and gardens. |

In Scotland and in the north of England, where the women work hard, in a few days after parturition they occupy themselves with the business of the house, and even very soon go out into the fields to work. The child is also carried out by another child, and is placed under a hedge or wall, and if the mother hears the child cry, she suckles it, or does so, from her belief of its wants. The food of the mother is ground oatmeal and milk, flour and milk, potatoes sliced and fried in fat." 142.

We believe, with Sir Astley, that nurses often eat and drink too much. Appetite suggests, habit continues the practice, and the constitution is frequently damaged by the repletion or intemperance that nursing generated. At the same time, there can be no doubt that lactation is debilitating, and that support is to some extent necessary. The amount must be determined by the constitution, the previous mode of living, &c. of the nurse.

It sometimes happens that the sexual secretion continues during lactation, and women have assured Sir A, that they and their children have been healthy. A woman who suckled sixteen months had the menstrual secretion during the last seven, yet her milk was abundant and the child healthy. These, however, must be considered as exceptions to general rules, for usually, if menstruation occurs during lactation, such a change is produced in the child's health and bowels, that a medical man is led to ask if the secretion has not returned; the woman also suffers from the great call upon her constitution which this double secretion produces, from the difficulty of supporting both at the same time.

Sir Astley speaks fully, and his observations are both curious and important on the influence of mental and moral emotions on the milk. The depressing passions vitiate, or diminish, nay arrest it. The following are strong instances of the effects of fright. A nurse was hired, and in the morning she had abundance of milk, but having to go fifty miles to the place at which the parents of the child resided, in a common diligence, the horses proved restive and the passengers were in much danger. When the nurse, who had been greatly terrified, arrived at her place at the end of the journey, the milk had entirely disappeared, and the secretion could not be reproduced, although she was stimulated by spirits, medicine, and by the best local applications a medical man could suggest. A lady in excellent health, and a good nurse, was overturned in her pony chaise, and when she returned home, pale, and greatly alarmed, she had no milk, nor did it return, and she was obliged to wean her child.

*Influence of Medicines on the Milk.*—That medicines will act through the milk on the child is, of course, well known. Mercury does so—purgatives do so. The medicines which affect the child the least, are olive oil, castor oil, confectio sennæ, and extractum colocynthidis compositum. The saline purges are apt to influence the child's bowels, or, as the nurses express it, to go to the milk.

"Iodine has been found in the milk by many persons. Dr. Rees writes:— 'A woman in Guy's Hospital had been taking iodine for a fortnight three times per diem, with five grains of hydriodate of potash; her milk was tested with sulphuric acid and starch, and the strongest indications of iodine were obtained.'

From the researches of Chevallier, Henry, and Peligot, on the milk of asses, to whom various medicines were administered, it appears that distinct traces of many remedial agents were readily detected in the lacteal secretion. Of these,—

Common salt was detected in abundance.

Sesqui-carbonate of soda passed in great quantity into the milk, rendering it alkaline.

Traces of sulphate of soda, when administed in doses of about two ounces, were readily detected.

Sulphate of quinine, although administered in large doses, did not appear to pass into the milk.

Iodide of potassium was readily detected, when administered in doses of a drachm and a half.

Oxide of zinc, tris-nitrate of bismuth, and sesqui-oxide of iron, were readily detected in the milk, when these substances were administered to the animal; but no traces of alkaline sulphurets, salts of mercury, or nitrate of potass, could be detected even after the ingestion of these drugs in considerable doses." 149.

We have exhausted the space allotted to this account of the human breast, and have only to add, that the remainder of the work is taken up with a notice of the changes which the mamma undergoes in age—a description of the gland in the male—and a brief sketch of its comparative anatomy.

It is quite unnecessary for us to eulogise the volume. It bears in every page the impress of the care that has been expended on its execution, and is one of which the profession in this country should be proud. The plates are admirable. We cannot conclude without wishing Sir Astley Cooper many coming years of health, and usefulness, and dignity.

